# Synthetic biology: a personal perspective from the world of big energy

**DOI:** 10.1111/1751-7915.13463

**Published:** 2019-07-17

**Authors:** Jeremy Shears

**Affiliations:** ^1^ Shell Research Limited London UK

As a recent Royal Society report^1^ noted, synthetic biology is creating ground‐breaking new technologies and applications across a wide variety of industry sectors, from pharmaceuticals to energy. However, challenges remain to the wider industrial uptake of synthetic biology, including the need to demonstrate profitability, scalability, concerns over public acceptance and difficulties with the language and definitions used. Even though there remain unanswered questions that demand considerable effort in fundamental research, particularly in chemistry and biology, the report concluded that there is reason to be cautiously optimistic. Synthetic biology stands on the cusp of deployment: as the costs of processes fall, the moment where rapid growth in industrialisation will take place is nearly upon us.

Governments, too, recognise the potential for economic growth and employment that the emerging synbio industry offers. For example, a UK government‐endorsed roadmap for synthetic biology^2^ that has triggered more than £300 million of public investment since 2012. As Clarke notes in a review on the uptake of synbio by business,^3^ much of the commercialisation of synbio today is driven by start‐ups and SMEs, often spin‐offs from academia, since these organisations have the necessary dynamics to be able to attract the required skillsets.

However, a number of established companies possess research groups familiar with synthetic biology. My own company has a long history of pioneering research in the biosciences. As a fresh PhD graduate in Biochemistry in 1986, I joined Shell Research Limited in the UK to work on stereoselective bio‐transformations, using microorganisms or pure enzymes. I was privileged to have as one of my mentors Professor Sir John Cornforth.^4^ Sir John had been appointed co‐director of Shell's Milstead Laboratory of Chemical Enzymology by Lord Rothschild in 1962 and received the Nobel Prize for Chemistry in 1975 for his work on the stereochemistry of enzyme‐catalysed reactions at Shell. In subsequent years, I worked in a range of technology and commercial positions around the world in Shell's chemicals, fuels and lubricants business. Fast forward to 2007, Shell began investing in the development of Advanced Biofuels and I was involved in setting up new bioscience laboratories, first in the UK, then in Houston, Texas. It has been an honour for me to recruit some of the most talented bioscientists from around the world to work in these laboratories.

Much of my work is concerned with the energy transition to low‐carbon and renewable energy sources. The world needs to take an urgent action to tackle climate change. The Paris Agreement^5^ sets a goal of keeping the rise in the global temperature below 2° Celsius and to pursue efforts to further limit the temperature increase to 1.5°C. Shell supports this, and our ambition is to make sure the energy we sell is in tune with society as it moves towards that goal. Shell believes the world is likely to have to stop adding to the stock of greenhouse gases in the atmosphere by 2070. This is a state known as net zero emissions.

The biosphere takes on an increasingly important role in stabilizing the climate through the 21st century, both from its carbon storage potential and from its role in providing renewable feedstock options for chemicals and materials. Photosynthesis not only provides a mechanism to capture solar energy in molecular form, but also generates molecular building blocks for future bio‐manufacturing industries. We are exploring how the repid developments in the biosciences, and synbio in particular, could become instrumental in our endeavour to provide more and cleaner energy.

We conduct R&D both in our Houston laboratory and in conjunction with leading academic institutes and SME's around the world. This is a close collaboration: it is common for University researchers to spend significant periods of time in our own laboratories and Shell staff to work at universities. We believe this is essential not only for the academics to understand the commercial drives but also for Shell to understand where the cutting edge of the science lies in this fast‐moving field. Moreover, such intimacy also stimulates the innovation that is required to tackle one of the main global challenges facing mankind. Feedback from the academic researchers indicates that they too get significant personal development from the relationship in areas such a deeper understanding of the importance of engineering scale‐up, the relevance of robust industrial host organisms, as well as the overall economic considerations that a commercial entity needs to take into account.

It is gratifying to see tremendous strides being taken by other commercial entities too, both large and small, and by academia and research funding councils. I expect that synbio will drive considerable growth in enterprise and employment creation in the energy sector and now is an exciting time for early‐career bioscientists to enter the field. The energy industry is certainly one of the most dynamic and exciting places to work!

++++++++

## Conflict of Interest

None declared.

## Explanatory Notes for Online Version at www.theabsurdmicrobe.com

1 ‘Synthetic biology: does industry get it?’; conference held on 8 February 2017 at the Royal Society, London. URL https://royalsociety.org/-/media/events/2017/02/tof-synbio/synthetic-biology-post-conference-report.PDF.

2 ‘A synthetic biology roadmap for the UK’ (2012), URL https://www.gov.uk/government/groups/synthetic-biology-leadership-council#synthetic-biology-roadmap-for-the-uk-2012, accessed May 2019.

3 Clarke, L.J. (2018) Synthetic biology – pathways to commercialisation. *Engineering Biology* 3, 2–5.

4 Hanson, J. (2014) John Cornforth (1917–2013). *Nature*, 506, 35.

5 ‘The Paris Agreement’, URL https://unfccc.int/process-and-meetings/the-paris-agreement/the-paris-agreement, accessed May 2019.

